# Innovative Materials with Possible Applications in the Wound Dressings Field: Alginate-Based Films with *Moringa oleifera* Extract

**DOI:** 10.3390/gels9070560

**Published:** 2023-07-09

**Authors:** Roxana Gheorghita, Roxana Filip, Ancuta-Veronica Lupaescu, Monica Iavorschi, Liliana Anchidin-Norocel, Gheorghe Gutt

**Affiliations:** 1 College of Medicine and Biological Sciences, Stefan cel Mare University of Suceava, 720229 Suceava, Romania; roxana.filip@usm.ro (R.F.); ancuta.lupaescu@usm.ro (A.-V.L.); monica.iavorschi@usm.ro (M.I.); 2 Suceava Emergency County Hospital, 720224 Suceava, Romania; 3 Faculty of Food Engineering, Stefan cel Mare University of Suceava, 720229 Suceava, Romania; g.gutt@fia.usv.ro

**Keywords:** biopolymers, antioxidant, antibacterial, essential oil

## Abstract

For a long time, biopolymers have proven their effectiveness in the development of materials with various applications, lately those intended for the biomedical and pharmaceutical industries, due to their high biocompatibility and non-toxic, non-allergenic, and non-immunogenic nature. The ability to incorporate various active substances in this matrix has yielded materials with characteristics that are far superior to those of classic, conventional ones. The beneficial effects of consuming *Moringa oleifera* have promoted the use of this plant, from Ayurvedic to classical medicine. The addition of such compounds in the materials intended for the treatment of surface wounds may represent the future of the development of innovative dressings. This study followed the development of materials based on sodium alginate and moringa powder or essential oil for use as dressings, pads, or sheets. Thus, three materials with the addition of 10–30% moringa powder and three materials with the addition of 10–30% essential oil were obtained. The data were compared with those of the control sample, with sodium alginate and plasticizer. The microtopography indicated that the materials have a homogeneous matrix that allows them to incorporate and maintain natural compounds with prolonged release. For example, the sample with 30% moringa essential oil kept its initial shape and did not disintegrate, although the swelling ratio value reached 4800% after 20 min. After testing the mechanical properties, the same sample had the best tensile strength (TS = 0.248 MPa) and elongation (31.41%), which is important for the flexibility of the dressing. The same sample exhibited a very high antioxidant capacity (60.78% inhibition). The materials obtained with moringa powder added presented good values of physical and mechanical properties, which supports their use as wound dressings for short-term application and the release of embedded compounds. According to the obtained results, all the biopolymeric materials with moringa added can be used as dressings for different wound types.

## 1. Introduction

Nowadays, the benefits of using biopolymers in fields other than the food industry have promoted the development of biomedical applications of great interest. Due to their well-known properties, such as regenerability, non-toxicity, non-allergenicity, high biocompatibility, and non-immunogenicity, biopolymers are used in the medical industry to obtain wound dressings, in tissue engineering [1], in gene therapy [2], in regenerative medicine, in dental medicine [3], and, perhaps most frequently, as materials for the encapsulation of various active substances, such as medicines, probiotics, and other compounds with beneficial roles in health [4,5]. These substances have proven to be very effective, especially due to their ability to slowly release the compounds they contain. Among the majority of tested biopolymers, sodium alginate is more intensively used due to its characteristics; its good physical, mechanical, and optical properties; and, above all, its durability and high solubility. Due to its biochemical characteristics (antioxidant and antimicrobial), it may be suitable as wound-healing material. According to recent research, alginate promotes the wound-healing process, collagen deposition, epithelial recovery, vascular density, and fibroblast proliferation [6,7].

*Moringa oleifera* (*M. oleifera*) is an important natural polysaccharide source; it is known as the “drumstick tree” or “horseradish tree” and is very popular in Asia and Africa because of its medicinal and nutritional value [8]. *M. oleifera* leaves have been intensively used since ancient times for their good biological activities, such as their antioxidant, antidiabetic [9], immunomodulatory, ethnopharmacological, and pharmacological effects (enhancing intestinal mucosal barrier properties, improving the activity of some digestive enzymes, and presenting prebiotic properties, and facilitating the slow release of drugs) [10], as well as for their capacity to cure different diseases (high blood pressure, hypercholesterolemia, cancer, inflammation, and diabetes) [11,12,13]. In African countries, they have been used to cure diabetes, flu and sinusitis fever, malaria, abscess, dysmenorrhea, articular pains, cancers, dysentery, eyesight problems, anemia, headaches and migraine, gonorrhea, indigestion, otitis, pulmonary troubles, prostatitis, stomach-related problems, swelling, varicella, tooth decay, sexual dysfunction, and oligospermia [14].

The antioxidant and antimicrobial activities of this plant have been intensively studied due to the promising results obtained. Good antimicrobial activity against *Staphylococcus aureus* and *Candida albicans* has been highlighted in different research studies; all the plant parts had better antioxidant activity than ascorbic acid, which was used as a reference compound [15]. A 12.5% minimum concentration completely inhibited *Staphylococcus aureus*, *Escherichia coli*, *Klebsiella pneumoniae*, *Salmonella typhi*, *Pseudomonas aeruginosa*, and *Shigella flexneri* strains [16].

*M. oleifera* has been tested as a promising antiviral agent, and according to studies that have been carried out since 1998, this plant possesses remarkable inhibitory activities against viruses like HIV *human immunodeficiency virus* (HIV), *herpes simplex virus* (HSV), *hepatitis B virus* (HBV), *Epstein–Barr virus* (EBV), and *Newcastle disease virus* (NDV) or *avian avulavirus 1*) [17]. In 2021, Xiong Y. et al. [18] reported that *M. oleifera* leaves had good antiviral activity against H1N1 in RAW264.7 cells; they could inhibit RBC hemolysis and plaque in H1N1-infected cells and block virus amplification, replication, and virulence. Surprisingly, due to its high content of apigenin, which is considered a future green natural antibiotic compound, Moringa is effective in hindering Mpro, the main targeted protease of SARS-CoV-2 [19].

Lower concentrations of the plant (25 µg/mL) were effective and against *Enterococcus faecalis* with no toxicity. According to the results, *M. oleifera* had an antibacterial effect during the first 48 h [20]. The antiviral potential of *M. oleifera* was also used for phytosanitary purposes, inhibiting eleven strains of *Erwinia amylovora* (EA), a phytopathogenic bacterium that affects *Rosaceae*. Its bacteriostatic activity resulted in an 80% reduction in film formation using low concentrations (1 mg/mL) [21]. *M. oleifera* has been used in disease management and as a plant biostimulant due to its composition of phytohormones, vitamins, mineral nutrients, phenols, flavonols, sterols, tannins, and other phytochemicals with beneficial effects by reducing the level of reactive oxygen species, lipid peroxidation, and electrolyte leakage [22].

The medicinal properties of *M. oleifera* are also significant and include a broad range of pharmaceutical activities and antimicrobial, antioxidant, antidiabetic, and anti-immunomodulatory properties. Common medicinal uses include the treatment of fever, eye diseases and head complaints, skin tumors, disorders or irritations, inflammation, bacterial infection, the common cold, external sores/ulcers, throat infections, muscle diseases, tumors, and cholera [23,24,25]. Its high anti-inflammatory activity can be helpful for wound healing, as it can protect and repair damaged tissue. It has been used as an effective therapeutic treatment against skin cancer, with chemo-preventive attributes, reducing tumor size when tested in vivo (mice) [26]. Pagano, C. et al. demonstrated that *M. oleifera* leaf extract was effective as a wound treatment due to its ability to stimulate cell growth and decrease the wound area after 12 h and completely close it within 24 h after exposure [27]. According to Chin et al. [28], films with *M. oleifera* possess optimal physicochemical properties for use as wound dressings, with good bioactive compound release properties and good proliferation of human dermal fibroblast cells and human keratinocytes. Good release properties were highlighted by Chin et al. [29] in their study, as well. According to their results, when moringa was added in order to develop nanofiber films, the loaded drug (vicenin—2) was released 2 h after exposure. Tested on albino rats, the leaf extracts minimized scar regions and promoted the healing process by suppressing anti-healing agents [30]. Al-Ghanayem et al., in their study, highlighted that local application of skin tissues with the plant extract promoted the wound-healing process without any skin irritation [31]. Due to its extensive local applicability and the possibility of processing, it can be used in dressings, pads, and sheets, as well as for medical, pharmaceutical, and cosmetic purposes. In the cosmetic industry, due to its properties, it can also be used as an antiaging agent, with oleamide, hydroxy nonadecatetraenoic acid, and quinic acid-O-pentoside being metabolites strongly correlated with antiaging activity, and caffeic acid inhibiting aging enzymes, such as collagenase, elastase, and hyaluronidase [32]. When used as a topical cream, it has reduced skin erythema and enhanced skin hydration levels without affecting melanin content and skin viscoelasticity [33]. Possessing an analgesic effect, the methanol extract of *M. oleifera* has produced significant local anesthetic activity when tested in vivo (on frogs and guinea pigs) [34].

Since 2012, 25 human interventional clinical studies have been carried out, but many of them have followed the consumption of *M. oleifera* in the form of drugs or supplements, as well as an addition to mouthwash for the health of the oral cavity. From what we know so far, there have been no studies involving the use of dressings with such additives [30].

The bioavailability of this plant’s chemical composition is higher than other plants due to the fact that in moringa most of the compounds exist in glycosylated form, which is known to be absorbed into epithelial cells faster than alkylated form [35]. According to various studies, the beneficial effects of using moringa are also due to the extraction method, with ultrasound-assisted extraction being the method that best preserves the bioactive compounds from this plant, especially those from the leaves [11,36,37]. Human studies conducted so far have not reported any adverse effects from using or consuming *M. oleifera* [27], and therapeutic consumption is safe at doses below 1000 mg/kg [38].

In the 21st century, medical resources in diagnosis and treatment have experienced huge developments. The objectives of modern patient care are focused on the fast and correct treatment of conditions, a reduction in the number of hospitalization days and costs, the quick return of patients to their daily activities, and a reduction in disease rates. The development of telemedicine and treatment alternatives represents a goal in the healthcare field [39,40,41].

The current study proposes the development of last-generation dressings, based on biopolymers, with the addition of *Moringa oleifera*, a plant well known for its regenerative properties since ancient times. Although global research in this field is becoming more and more evident, in Romania, the pharmaceutical market has not met such challenges. Also, from meta-analysis, it can be concluded that the regenerative properties of this plant have been studied when it was applied either locally or embedded in classic materials/conventional dressings. The development of a wound dressing based on biopolymers would represent a step forward; according to experience in the field, biopolymers are substances with beneficial effects on health, with the ability to retain natural or synthetic compounds in their matrix and with delayed release. Our results, presented in this paper, support the use of sodium alginate and *M. oleifera* as dressings for pharmaceutical or cosmetic use. Further determinations include in vivo application and cytotoxicity evaluation, wound models, and safety assessments. According to other results presented in the literature, this plant is one of the best options regarding natural materials with regenerative properties, being the most used compound for wound healing compared to the topical usage of other extracts [42].

## 2. Results and Discussion

Our study aimed to identify the possibility of incorporating moringa oleifera into biopolymer matrices used for the development of wound dressings for surface wounds. Thus, the results obtained from the evaluation of the microstructure and the physicochemical and mechanical properties are presented in the following figures and tables.

### Microstructure

**Figure 1:**
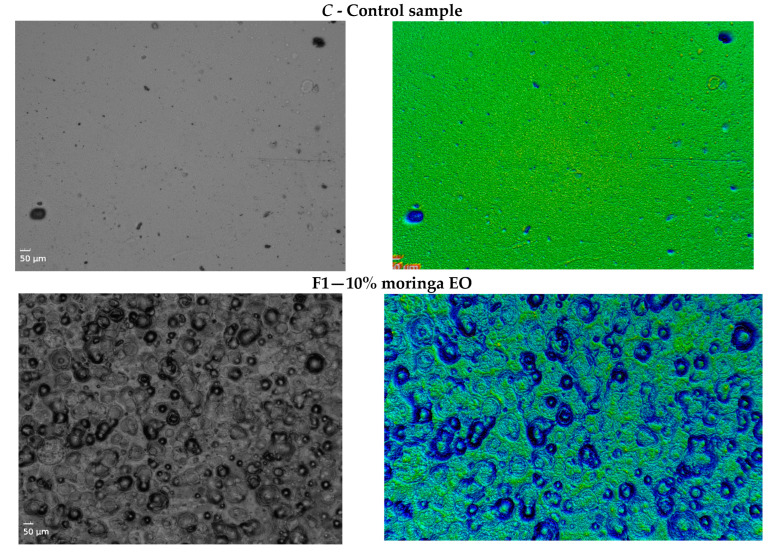

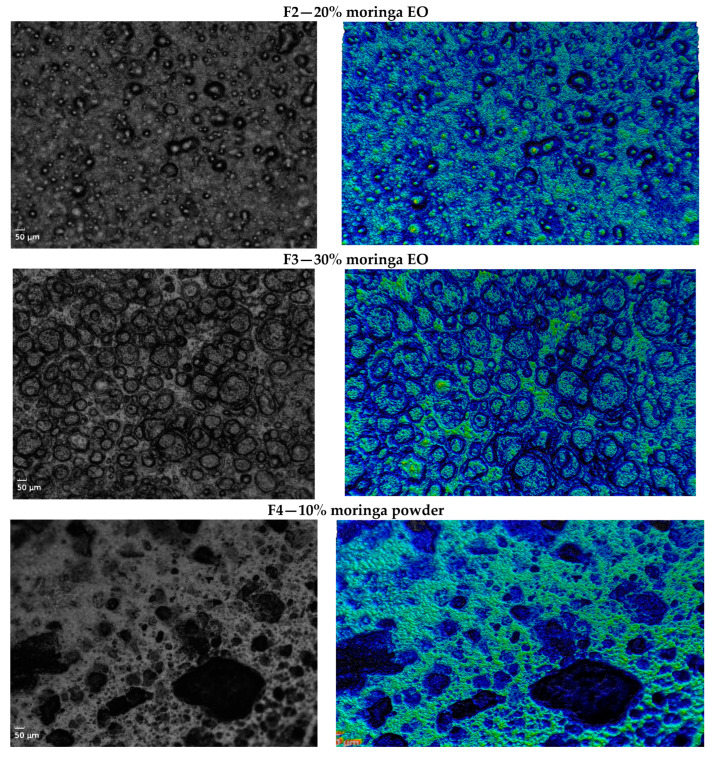

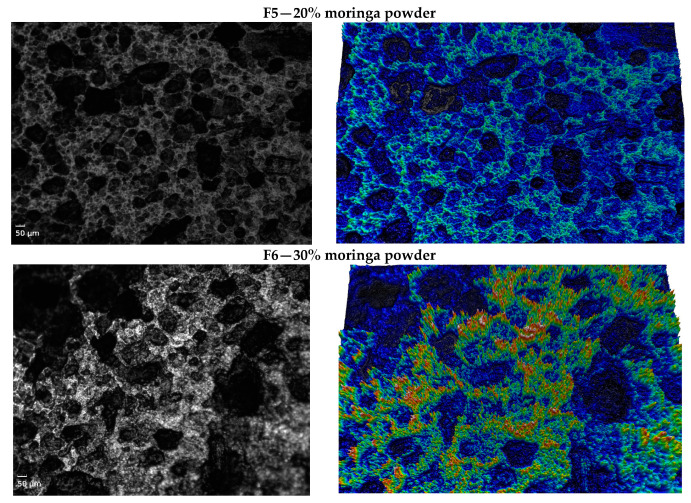
Microstructures and microtopographies of samples.

The microstructure of the materials was influenced by the additives. According to the images from Figure 1, the control sample, without *M. oleifera* in its composition, has a smoother surface without pores. The microstructures of the samples with essential oil (F1, F2, and F3) highlight the need to use an emulsifier, such as Tween 80, in order to better homogenize the film-forming solution. This aspect can be a beneficial one if we take into account the ability of the matrix to retain these particles, which is a favorable aspect for the development of materials with various encapsulated additives. The same pattern was observed by Kamel et al. when they developed sodium-alginate-based materials with *M. oleifera* leaf extracts in their composition [43]. Thus, the material morphology was not affected by an increased level of plant composition in the matrix.

The samples with the addition of plant powder (F4, F5, and F6), although they showed better homogenization, contained pores in the structure. Increasing the plasticizer content in the composition, whether it is glycerol or even water, can develop materials with a more compact structure.

The thickness of the films was strongly influenced by the addition of essential oil and moringa powder. Thus, if the thickness of the control sample was approximately 70 µm, the value increased directly proportional to the percentage of moringa addition. The samples with moringa powder (F4–F6) showed much higher values than those of the control sample. The thickness of sample *F6*, with the addition of 30% moringa powder, was 200% higher than that of the control sample. Comparing the results obtained with those presented by Rodriguez, G. et al. [44], the thickness of the samples with sodium alginate and moringa was much lower than that of the films developed from papaya and moringa. According to the results presented by them, the films with moringa showed values of 0.11 mm, distinguishing them from those with moringa and ascorbic acid, when the thickness was 0.28 mm. The same pattern was observed by Braham F. et al. for methylcellulose films with the addition of *M. oleifera* when the medium thickness was 190 µm [45]. According to these results (Table 1), sodium alginate may be a more suitable biopolymer for the development of such dressings, taking into account the fact that, at this moment, classical dressings are of reduced thickness for the comfort of the patient.

The density of the samples decreased with the increase in the essential oil in the composition and increased with the addition of moringa powder. The density of the control sample was 1.52 g/cm^3^, with similar values found in the literature [28].

The roughness of the films was higher for samples C, F2 (addition of 20% moringa essential oil), and F6 (addition of 30% moringa powder). According to the results, no correlation could be made between roughness and the addition of oil or moringa powder, but it could be observed that they changed the microstructure of the materials, as can be seen in Figure 1.

The opacity was greatly influenced by the additives. Thus, it increased directly proportional to the addition of essential oil or powder in the composition, being approximately six times higher in the case of the sample with 30% powder (F6) compared to the control sample (*C*). A deviation could be observed for samples F2 and F3 when a larger volume of essential oil in the composition facilitated the development of a film with lower opacity and vice versa.

The mechanical properties were greatly influenced by the addition of moringa. The materials had better tensile strength with the addition of oil, increasing from 0.174% (F1, with the addition of 10% oil) to 0.248% (F3, with the addition of 30% oil). Similarly, the elasticity of the samples also increased; the addition of 30% oil facilitated the development of a material twice as elastic as F1 (31.41%—F3 compared to 17.15%—F1 sample). When sodium-alginate-based films with moringa in their composition were tested, Chin et a. observed that the best value of TS was higher than the F3 values (10,799 MPa) with a higher percentage of elongation at 35.6% [28]. According to Ningrum et al. [46], the addition of *M. oleifera* leaves to the film composition decreased tensile strength and reduced flexibility. In this case, the addition of graphene oxide to the composition improved the mechanical properties.

These results are encouraging for the development of materials with added moringa essential oil.

The addition of moringa powder in proportions of 10 and 20% led to the development of samples that were stronger in terms of tensile strength but also less elastic than the control sample. However, according to the results, a 30% addition of powder negatively influenced resistance to breaking and elasticity, with the samples being even weaker than the control.

The luminosity of the samples was not influenced by the addition of essential oil, with the results being similar to those of the control sample (Table 2). On the other hand, the luminosity of the samples with the addition of moringa powder was much lower, which was due to its dark-green color. Overall, the luminosity values of the F4–F6 samples were higher than that of moringa powder (41.24). The differences between the control and F1–F6 samples are noted with Δ*E*_1_ in Table 2. On an ordinary scale, ΔE values range between 0 and 100. Human eyes cannot observe variations if Δ*E* is lower than 1, although the differences are perceptible through close observation when Δ*E* = 1–2, perceptible at a glance (Δ*E* = 2–5). When Δ*E* has values larger than 5, two different colors can be observed [47].

In our case, obvious differences can be observed in samples with moringa powder in their composition (F4, F5, and F6) and are exactly opposite between gel and film (higher values of ΔE_2_). The differences in the a* (the color in the green–red field) parameter can be clearly observed between gel and film samples, with those of gels being lower than 2 value, regardless of the moringa type added (essential oil or powder). According to the negative a* values presented in Table 2, the color of the films is greener than the gels (the color of which tends to be red). The control sample, without any addition, presents a lower a* value (−5.82), highlighting the fact that the negative values of the a* parameter are more influenced by the film-forming solution materials (biopolymers, plasticizer, or water) than by the additives. The parameter values of b* are significantly higher in the gel samples than in the films. For example, the b* value difference between film and gel, for the *F6* sample, is 19.91, even if the moringa powder has a b* value of 17.99. To conclude, the films present a greener–bluer color compared to the gels.

**Figure 2:**
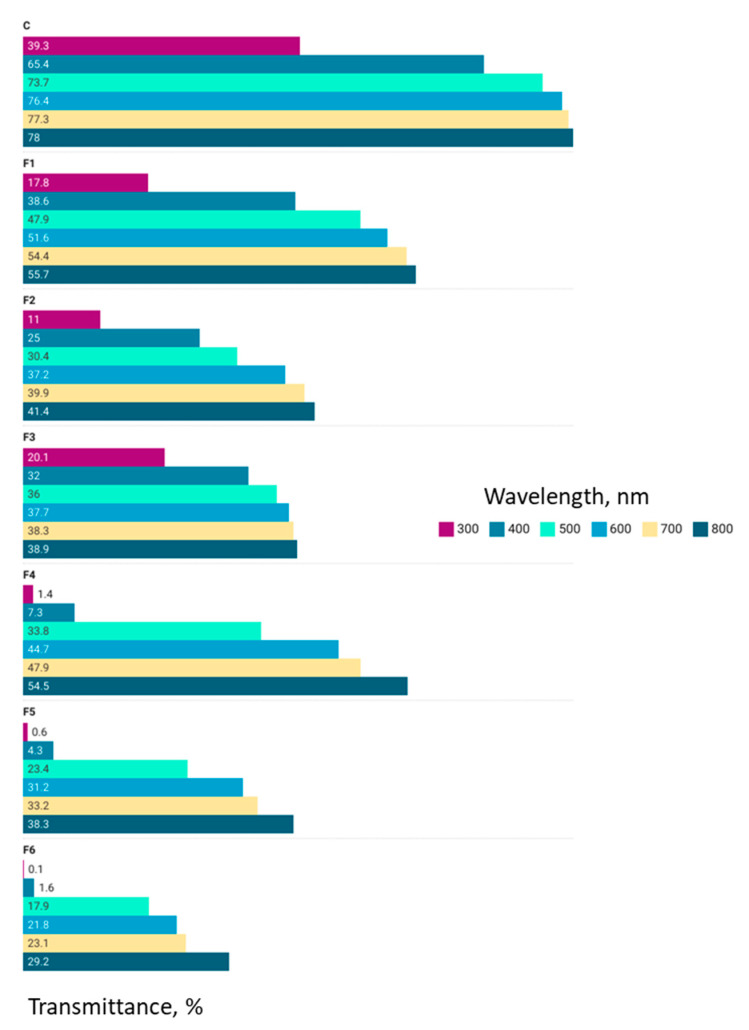
Transmittance variation at 300–800 nm wavelength.

For all tested samples, the lowest transmittance values were identified at a wavelength of 300 nm, and the highest were identified at 800 nm (visible light). The highest transmittance values, regardless of the tested wavelength, were identified in the control sample (Figure 2). Regardless of the type of additive, whether it was oil or powder, the materials were more resistant to the action of UV rays due to the reduction in transmittance values, thus being able to protect against the degradation or oxidation of the constituent compounds. The transmittance values were inversely proportional to the addition of essential oil or moringa powder, decreasing in the order of 30% addition (F3 and F6) < 20% addition (F2 and F5) < 10% addition (F1 and F4).

The water activity index shows low values (below 0.4), which indicates the ability of the material to prevent the development and proliferation of microorganisms, as well as the possibility of immediate hydration after immersion in water at room temperature (Table 3). In this way, the compounds existing in the matrix of the material can be released and absorbed much faster and more efficiently than those existing in classic, conventional materials.

**Figure 3:**
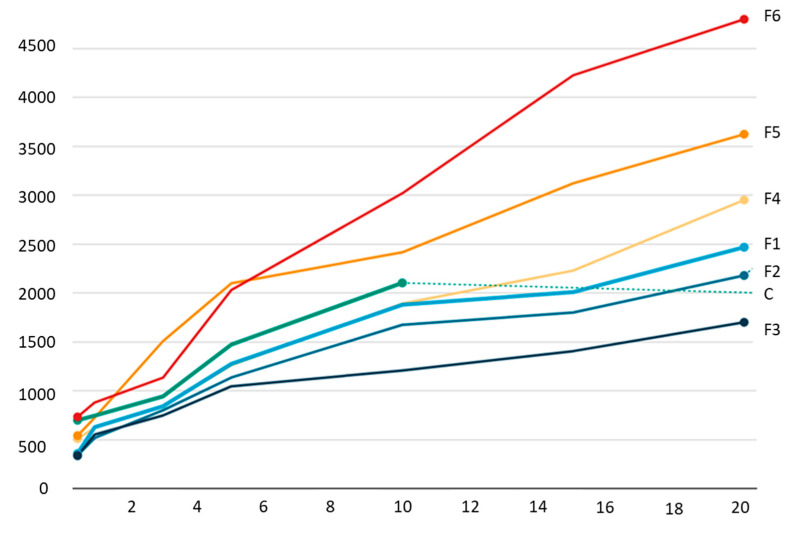
Swelling ratio of tested samples (X-axis represents the SR value, %; Y-axis represents time, min).

All tested samples presented a high swelling ratio index, highlighting the hydrophilic nature of the materials, regardless of the additives in the film-forming solution. As expected, the samples with moringa essential oil in the composition presented lower values for the swelling ratio index, taking into account the hydrophobic nature of the added oil (Figure 3).

The control sample, without the addition of powder or oil in its composition, was completely solubilized after 10 min of keeping it in water at room temperature. The fact that the other samples resisted immersion in water for more than 20 min indicates that these materials can be used as dressings and can release the substances they contain for a long time. To facilitate this process, it is recommended that the materials are initially moistened and then placed on the affected area to facilitate the release of the active compounds embedded in the film matrix.

**Figure 4:**
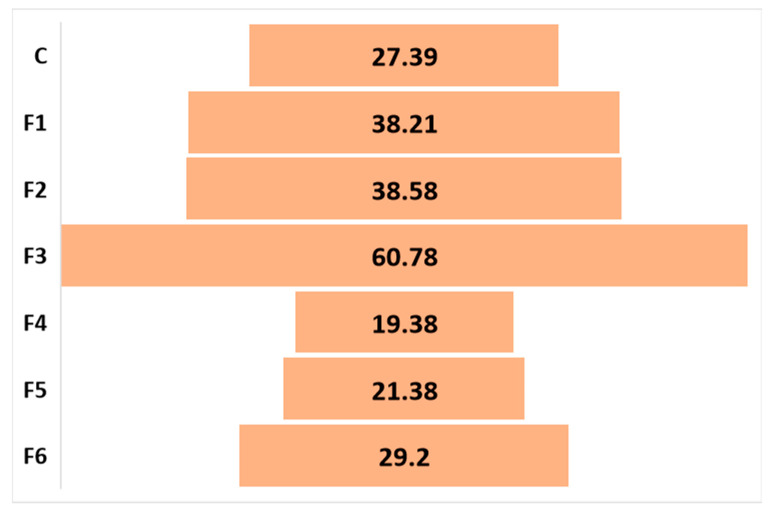
Antioxidant activity of biopolymeric films (% inhibition) (X-axis—the tested samples; the values are presented in the graphic).

The results in Figure 4 indicate that moringa is an important source of antioxidants and that the material development process did not have a negative influence. According to the results, the films with moringa essential oil in the composition showed higher antioxidant activity (60.78% for samples with 30% moringa essential oil added), unlike those that contained the powder from the leaves of the plant (maximum 29.2% for samples *F6*, with 30% moringa powder). If we take into account the fact that the control sample, without the addition of moringa in its composition, presented a value of 20%, we can consider the fact that only the essential oil of moringa, added to the composition of the film-forming solution, brought important antioxidant intake. Moreover, according to studies carried out by Penalver et al. [48], the antioxidant activity of moringa leaf powder, tested using the ABTS method, was 41.40%. The antioxidant activity of the control sample may be due to the sodium alginate content, known for its antioxidant properties. However, the studies showed much lower results than those obtained in this case (below 10% in a study developed by Dou L. et al. [49]). In their paper, Kim, D.-S. et al. presented a better antioxidant activity of *M. oleifera* (72.89%) and highlighted the fact that antioxidant activity is higher when it is used a non-polar extraction solvent compared to a polar one [50].

The higher antioxidant effect of *M. oleifera* can be attributed to the free hydroxyl compounds that prevent oxidation activity [15]. Furthermore, the antioxidant activity is potentiated by the high flavonoid and polyphenol contents [51], as well as the antioxidant compounds, such as myricetin and quercetin, mainly found in the leaves. Hydroxybenzoic acid derivatives, such as gallic acid, protocatechuic acid, syringic acid, and gentisic acid, as well as hydroxycinnamic acids, isoquercetin, rutin, catechin, vanillin, resveratrol, naringenin, cryptochlorogenic acid, and catechin have potential antioxidant and antimicrobial activity [52].

**Figure 5:**
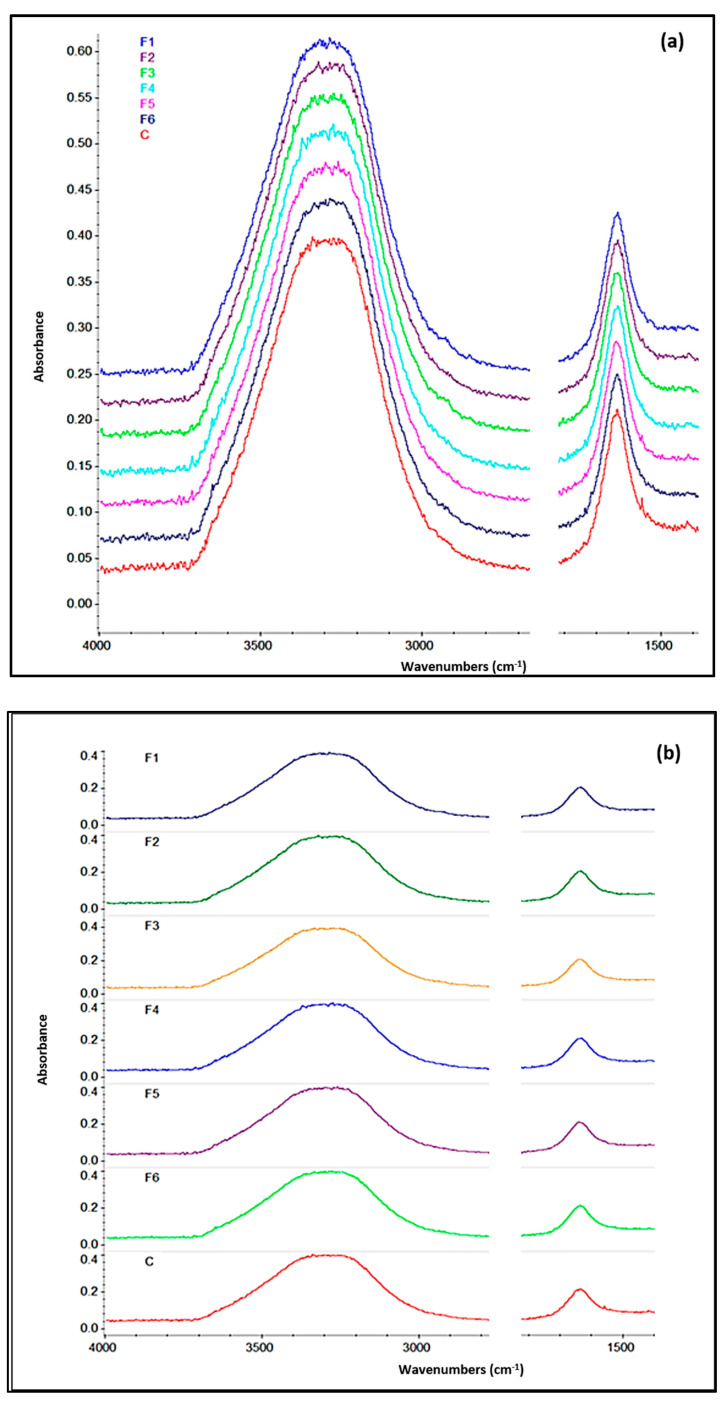
FTIR spectra of control (C) and samples with essential oil (F1, F2, F3) or addition of moringa powder (F4, F5, F6) stacked or overlaid (**a**,**b**).

FTIR spectroscopy was performed to reveal either the formation of new chemical bonds or the alteration of existing ones. In principle, if, by comparing the spectrum recorded for the control sample with that of the newly created samples (Figure 5), changes in the absorption maxima of the peaks are observed, they can be attributed to a possible interaction between the alginate and essential oils used, which leads to new bonds and eventually new vibrational bands. However, although the analyzed samples contained various amounts of *M. oleifera* extract, no structural differences were registered in the FTIR spectra, which highlights the stability of samples and integrity of chemical bonds. Thus, all the spectra recorded an intense peak in the range of 3000 to 3600 cm^−1^ characteristic of the alginate hydroxyl bond (-OH) and another strong signal in the range of 1500–1700 cm^−1^ corresponding to asymmetric stretching vibration of the alginate carboxylate groups (COO-) [53,54]. Therefore, it can be concluded that the alginate found in the structure of the films does not interact chemically with the essential oils due to the similarities found between the recorded IR spectra of the analyzed samples and the control. This confirms the fact that the essential compounds were incorporated inside the films through physical and not chemical interactions, thus highlighting their chemical stability vital for biopharmaceutical applications.

## 3. Conclusions and Future Aspects

The main goal of this research was to develop biopolymer materials that can be used as dressings in the pharmaceutical or biomedical industry, especially due to their high biocompatibility, as well as non-toxic, non-allergenic, and non-immunogenic characteristics. The addition of *M. oleifera* in such materials can only be extremely beneficial, taking into account its characteristics and its wide use in the treatment of various pathologies. The strong antioxidant and antimicrobial characteristics promote its use in such materials, which can be useful for tissue regeneration, stopping bleeding, and treating scratches or superficial wounds. For this purpose, materials based on sodium alginate, with the addition of moringa powder or essential oil were developed. The samples with the highest moringa oil concentration added to their composition presented the best mechanical characteristics (TS = 0.248 MPa, E = 31.41%) and antioxidant activity (60.78%). The solubility of the samples is higher in the case of those with moringa powder in their composition. This can be explained by the hydrophobic nature of the oils. The swelling ratio of the sample containing 30% moringa powder was the highest (4800%) in contrast to the volume of water absorbed by the samples containing 30% essential oil (1700%). Even if the SR value was high, the samples kept their shape, even after 20 min of immersion. This property of the material is beneficial for the development of dressings that can be used for a longer period of time without degradation.

Thus, for use as dressings, depending on the purpose and destination, the composition may contain a higher amount of moringa powder if it is intended to dissolve them more quickly or, similarly, essential oil added if it is intended that the product lasts for a longer period and allow the prolonged release of the incorporated compound. The microtopography of sample F3 indicates that the matrix of the material is homogeneous, with fat-soluble globules well embedded into the structure, which highlights the possibility of using this composition in order to encapsulate various types of natural compounds with a role in epithelial regeneration.

According to the results obtained, the addition of moringa essential oil, in any proportion, is recommended to the detriment of the powder. The latter can be used for food supplements, while the oil can be applied topically, being more suitable for such materials. Future aspects may include in vivo testing using human cell lines in order to evaluate the biocompatibility of these materials and the time of action.

## 4. Materials and Methods

### 4.1. Materials

All substances used were analytical-grade, non-toxic, allergenic, and highly compatible. Sodium alginate and glycerol were purchased from Sigma Aldrich Company, and moringa powder from leaves was purchased from Organic India, Ltd. (Lucknow, India), certified organic by Control Union (CU 801983), and produced and processed according to NPOP Standard of India and EC Regulation. According to the manufacturer, 2 g (1 teaspoon) of moringa powder can be ingested twice a day and is safe for long-term use. A portion of moringa contains a significant number of amino acids, as shown in Table 4. Besides these, specialized studies have highlighted the fact that it also contains an important amount of fiber, calcium, potassium, magnesium, iron, phosphorus, and copper, as well as vitamins (A, B1, B2, B3, C, and E), chlorophyll, and adequate sources of phytochemicals, such as phenolic acids, flavonoids, tannins, saponins, and alkaloids [13].

Cold-pressed moringa seed oil was purchased from CrisNatur Romania (Sibiu, Romania) and contained 100% moringa essential oil.

### 4.2. Methods

Films were developed through casting method; thus, 2.8 g of sodium alginate, glycerol, *M. oleifera* essential oil or powder were dissolved in 90 mL water, stirred under homogenization at 550 rpm (DLAB MS H280-Pro, Qing Dao, China), and maintained for 25 min at 80 ± 0.8 °C. After that, the film-forming solution was poured into a silicone support until completely dry (20–24 h). The film compositions are presented in Table 5.

The film thickness (*t*, µm) was taken into account after five readings for different areas of the material surface. For this purpose, a Yato electronic micrometer (Shanghai, China) was used, with a precision of 0.002 mm. For the calculation of the retraction ratio (*RR*, %) values, the thickness of the film-forming solution poured on the silicone support immediately after development (*T1*, 200 µm) and the final thickness of the film (*T2*, µm) were taken into account. Thus, the result was calculated using the following formula:

(1) $$RR=\frac{T1-T2}{T1}\times 100$$

The density (*D*, g/cm^3^) of the films was determined by relating their mass (*w*) to the thickness (*t*) and surface (*a*) [55]:

(2) $$Density=\frac{w}{a\times t}$$

Transmittance (*T*, %) and absorbance (*A*) were read spectrophotometrically (Epoch, BioTek Instruments, Winooski, VT, USA) using 1 cm × 3 cm film samples. The transmittance was read at a wavelength range of 300 to 800 nm and absorbance at 600 nm. An empty cuvette was used as standard. The determinations were made in triplicate. The opacity of material (*O*, A/mm) was calculated according to the following formula:

(3) Opacity=A/t

where *A* is the absorbance and *t* is the thickness (mm).

The water activity index (*a_w_*) was determined with AquaLab 4TE equipment (Meter Group, München, Germany) at 23 ± 0.3 °C. The results indicate the average of five readings of different areas of tested materials. The evaluation of this parameter is of interest when dehydration of the films occurs. A low water activity index value prevents the development and proliferation of microorganisms. According to the literature, a water activity index value above 0.7 is required in order to survive environmental conditions.

The samples’ (film-forming solution and membranes) colors were evaluated using the CIELab system with a Chroma Meter CR400 colorimeter (Konica Minolta, Japan). The results represent the average of ten readings made on the different areas of the entire material surface. In order to test the color difference between the samples tested before and after storage, the color variations (Δ*E*) were calculated. A blank standard illuminant was used, with L* = 94.12, a* = −5.52, and b* = 9.27.

(4) $$\Delta E=\sqrt{{\left(\Delta L^{*}\right)}^{2}+{\left(\Delta a^{*}\right)}^{2}+{\left(\Delta b^{*}\right)}^{2}}$$

where ΔL*, Δa*, and Δb* are the differentials between the sample and control color parameters, between gel and films, and between materials color (essential oil or powder).

In order to achieve a better evaluation of color changes during development, Δ*E*_1_ and Δ*E*_2_ were calculated. Δ*E*_1_ represents the color differences between gel samples (film-forming solution before drying, immediately after casting on the silicone surface) and films (final product). Δ*E*_2_ represents the color differences between essential oil/moringa powder (raw material) and films. Our aim was to determine and observe the color differences between essential oil/moringa powder raw material—gels obtained—films. According to our results, differences between those samples could be seen. Taking into account these determinations, at an industrial scale, the producer can be aware of the fact that product color will change, so they can adjust the final color according to initial color parameters.

The microstructure of images was observed with a Celena Microscope, and images and microtopographies were analyzed using Mountains Premium 9 software (Digital Surf, Lavoisier, France).

The tensile strength (*TS*, *MPa*) and elongation (*E*, %) were tested with ESM Mark 10 texturometer, according to STAS ASTM D882 (Standard Test Method for Tensile Properties of Thin Plastic Sheeting) [56] and were calculated according to Formulas (5) and (6) [57]. As such, a 5 KN cell and special grips for thin films and foils were attached, and 1 cm × 10 cm film samples were tested. The travel speed was set at 10 mm/min, and the working temperature was 25.6 ± 0.7 °C.

(5) $$TS=\frac{F}{a}$$

where *F* is the maximum load (kN) and *a* is the surface (mm^2^). The travel speed of 10 mm/min was chosen based on standard requirements for testing films and foils of 5 to 10 mm/min, as well as on the published evidence [58].

The elongation at break (*E*) represents the ratio between final length (Δ*l*) and initial length (*l*) after test specimen breakage.

(6) $$E=\frac{\Delta l}{l}\times 100$$

When the developed samples are intended to be used as materials that release compounds from the matrix after immersion in water, it is very important to test their solubilization and swelling capacity. Thus, in addition to the determinations regarding moisture content, the swelling ratio and solubility in water were also monitored.

Thus, for moisture content analysis (*MC*, %), 3 cm × 3 cm film samples were weighed before and after storage for 24 h at a temperature of 110 ± 2 °C. The determination was made in triplicate, and the result was calculated with the following formula:

(7) $$MC=\frac{W0-W1}{W0}\times 100$$

where W0 represents the sample mass before drying (g) and *W1* represents the dried mass (g).

In order to evaluate the solubility, the swelling ratio index (*SR*, %) was taken into account. This evaluation is important when the material is intended to be use in biomedical applications for dressings or other sheets and must facilitate the release of compounds from the matrix and dissolve together with them. In this way, the skin can absorb the entire amount of incorporated substances, which can be more easily assimilated than those existing in classic dressings, which are often absorbed by the material used as a support. For determination, 3 × 3 cm film samples were weighed (*M0*), immersed in water at room temperature (21 ± 0.6 °C), and maintained for 0.5, 1, 3, 5, 7, 10, 15, 20 min. Then, the samples were removed from the container, the excess water was eliminated using filter paper, and the samples were re-weighed (*Mt*). The determinations were made as a single experiment.

The final value of swelling capacity was calculated with the following formula:

(8) $$SR=\frac{Mt-M0}{M0}\times 100$$

Antioxidant assessment was performed using ABTS radical scavenging capacity measurement, according to the method described by Xu et al. [59] with some modifications. Briefly, the film sample was cut into 20 mm × 20 mm. ABTS radical solution was prepared by gently mixing 10 mL of 7 mM ABTS solution and 10 mL of 2.45 mM potassium persulfate solution. Afterward, the reagent was incubated for 16 h in the dark at room temperature to allow the generation of the ABTS radical. Before use, the reagent was brought to an absorbance of 1 by diluting with ethanol. The analysis method involved immersing the films in 2 mL ABTS reagent and incubating them in the dark for 0.5/1 h at 37 °C, 500 rpm. Finally, the absorbance was read at 734 nm using an Epoq spectrophotometer (BioTek Instruments, Winooski, VT, USA). The experiment was carried out in triplicate, and the radical scavenging activity (%) was calculated according to Formula (8), where *A*0 is the absorbance of the control sample, and *A*1 is the absorbance of the sample:

(9) $$Inhibition=\frac{A0-A1}{A0}\times 100$$

The FT-IR spectra of samples were achieved with an FT-IR spectrometer (Thermo Scientific, Karlsruhe, Dieselstraße, Germany) with ATR IX option. The results were obtained within a range of 400–4000 cm^−1^ with a detector at 4 cm^−1^.

## Figures and Tables

**Table 1:** Physical and mechanical properties.

Sample	Thickness, µm	Retraction Ratio, µm	Density, g/cm^3^	Roughness, nm	Opacity, A/mm	Tensile Strength, MPa	Elongation, %
C	69.40 ± 2.01	65.30 ± 1.50	1.52 ± 0.03	1.29 ± 0.04	0.79 ± 0.33	0.125 ± 0.06	14.22 ± 0.74
F1	86.20 ± 2.23	56.90 ± 1.11	1.40 ± 0.12	1.32 ± 0.15	2.38 ± 0.96	0.174 ± 0.12	17.15 ± 0.03
F2	95.60 ± 2.80	52.20 ± 1.40	0.91 ± 0.24	2.14 ± 0.13	3.27 ± 1.30	0.206 ± 0.07	28.96 ± 0.14
F3	134.60 ± 2.06	32.70 ± 1.03	0.49 ± 0.03	1.62 ± 0.04	2.52 ± 0.95	0.248 ± 0.69	31.41 ± 0.66
F4	110.40 ± 2.33	44.80 ± 1.17	0.66 ± 0.14	1.16 ± 0.06	2.48 ± 0.97	0.166 ± 0.03	11.47 ± 0.92
F5	127.60 ± 2.15	36.20 ± 1.08	0.93 ± 0.06	1.32 ± 0.04	3.45 ± 1.31	0.138 ± 0.01	11.12 ± 0.30
F6	143.60 ± 2.41	28.20 ± 2.20	1.66 ± 0.02	2.97 ± 0.17	4.31 ± 1.61	0.104 ± 0.04	8.74 ± 0.19

**Table 2:** Color parameters and color differences between tested materials.

Sample	L*	a*	b*	ΔE_1_	ΔE_2_	ΔE_3_
C	89.53 ± 0.81	−5.82 ± 0.03	13.43 ± 0.20	-	60.02 ± 1.95	-
F1	89.05 ± 0.50	−5.71 ± 0.03	14.58 ± 0.15	1.47 ± 1.20	59.43 ± 0.94	63.98 ± 1.75
F2	88.25 ± 0.46	−5.57 ± 0.05	16.08 ± 0.78	3.16 ± 0.84	60.39 ± 0.53	63.40 ± 0.97
F3	90.25 ± 0.67	−5.81 ± 0.03	13.84 ± 0.22	1.47 ± 0.04	60.90 ± 0.92	65.49 ± 0.92
F4	71.73 ± 2.46	−5.12 ± 0.38	26.65 ± 0.87	22.29 ± 1.38	53.66 ± 0.77	33.10 ± 0.65
F5	53.62 ± 3.28	−1.71 ± 0.82	24.45 ± 0.55	37.94 ± 1.35	36.92 ± 1.07	14.73 ± 0.88
F6	48.92 ± 1.55	−1.36 ± 0.41	22.67 ± 0.63	41.93 ± 1.54	30.77 ± 0.85	9.24 ± 0.79
C gel	29.71 ± 0.92	−0.82 ± 0.17	6.80 ± 0.35	-	-	-
F1 gel	30.87 ± 0.27	−1.88 ± 0.02	3.31 ± 0.03	3.97 ± 1.60	-	9.89 ± 1.66
F2 gel	29.56 ± 0.06	−1.81 ± 0.03	2.82 ± 0.02	4.21 ± 0.95	-	9.67 ± 1.45
F3 gel	30.83 ± 0.08	−1.96 ± 0.02	3.66 ± 0.02	3.67 ± 1.11	-	9.53 ± 0.99
F4 gel	24.03 ± 0.09	−1.47 ± 0.02	1.91 ± 0.02	7.59 ± 0.56	-	23.61 ± 1.76
F5 gel	24.55 ± 0.07	−1.60 ± 0.01	2.44 ± 0.04	7.22 ± 0.42	-	22.85 ± 0.78
F6 gel	25.24 ± 0.05	−1.59 ± 0.01	2.76 ± 0.07	6.14 ± 1.07	-	22.14 ± 0.30
Moringa powder	41.24 ± 0.51	−3.13 ± 0.05	17.99 ± 0.12	-	-	-
Moringa essential oil	25.41 ± 0.46	−0.53 ± 0.03	11.44 ± 0.54	-	-	-

L*—lightness value, a*—green-red axis, b*—blue-yellow axis, ΔE_1_—color difference between samples and control, ΔE_2_—color differences between gel samples and films, ΔE_3_—color differences between essential oil and film of moringa powder and films, respectively.

**Table 3:** Solubility properties of developed films.

Samples	MC, %	a_w_
**C**	16.82 ± 0.06	0.34 ± 0.004
**F1**	13.17 ± 0.21	0.38 ± 0.003
**F2**	11.14 ± 0.37	0.35 ± 0.004
**F3**	10.21 ± 0.05	0.34 ± 0.007
**F4**	15.12 ± 0.68	0.35 ± 0.015
**F5**	16.46 ± 0.03	0.35 ± 0.005
**F6**	18.17 ± 0.16	0.33 ± 0.002

MC—moisture content, a_w_—water activity index.

**Table 4:** The content of a portion of moringa (2 g) in amino acids (502.38 mg).

Alanine	Arginine	Aspartic acid	Cysteine	Glycine	Histidine	Isoleucine	Leucine	Lysin
31.70	31.56	54.62	5.18	26.28	12.54	23.62	43.66	29.46
Threonine	Tryptophan	Glutamic acid	Tyrosine	Valine	Methionine	Phenylalanine	Proline	Serine
24.44	12.08	70.90	17.62	29.88	8.30	31.96	25.14	23.44

**Table 5:** The composition of the films.

Sample	Alginate (g)	Glycerol (g)	Moringa EO (g)	Moringa Powder (g)	Water (mL)
C	2	0.8	-	-	90
F1	1.74		0.26	-	
F2	1.48		0.52	-	
F3	1.22		0.78		
F4	1.74		-	0.26	
F5	1.48		-	0.52	
F6	1.22		-	0.78	
